# Experimental Validation of a Novel Generator of Gas Mixtures Based on Axial Gas Pulses Coupled to a Micromixer

**DOI:** 10.3390/mi12060715

**Published:** 2021-06-18

**Authors:** Florian Noël, Claire Trocquet, Christophe A. Serra, Stéphane Le Calvé

**Affiliations:** 1 ICPEES UMR 7515, Université de Strasbourg/CNRS, F-67000 Strasbourg, France; noel.florian.gim@gmail.com; 2 In’Air Solutions, 25 Rue Becquerel, F-67087 Strasbourg, France; claire.trocquet@gmail.com; 3 Institut Charles Sadron (ICS) UPR 22, Université de Strasbourg/CNRS, F-67000 Strasbourg, France; ca.serra@unistra.fr

**Keywords:** gas generator, gas mixing, pulsed flow, micromixer, multi-stages, Benzene, aromatic compounds

## Abstract

In this work, a novel generator of gas mixtures previously numerically investigated and based on axial gas pulses coupled to a micromixer has been conceived, manufactured, and validated. Standard gaseous pollutant mixtures and pure nitrogen or pure air were introduced in a microdevice designed to generate alternating axial gas pulses which were downstream homogenized by means of a multi-stage modular micromixer. The dilution, and therefore the final pollutant concentration, was controlled by two parameters: the ratio between the times of each of the two gas pulses and the partial pressure of the pollutant(s) mixture added to the device. The gas mixture generator was coupled to an analyzer to monitor the concentration of aromatic pollutants. The response time was optimized to be lower than 2 min in accordance with the analytical instrument. The quantity of pollutants measured at the micromixer’s outlet increased linearly with the expected gas concentration of 3.7–100 ppb generated by this novel microfluidic generator and fitted perfectly with those obtained by a reference gas dilution bench. At 5 ppb, the precision on the concentration generated is close to that obtained with the conventional gas mixing bench, i.e., around 10%.

## 1. Introduction

The mixing of different compounds with a known and precise ratio is often of great interest in many areas. On industrial level, this is the case for mixtures involving solids, such as in agriculture [1,2,3], pharmaceutical and cosmetics industries [4,5,6,7], food industry [8,9] or the manufacture of certain materials such as ceramics [10,11]. Other applications involve mixing liquids, such as for the food industry [8,12], refining fuels when choosing octane level [13,14], or managing chlorine levels in swimming pools [15]. Finally, gas mixtures are also used in many fields, for example in the beer brewing process [16], for the generation of anesthetic gases [17,18] and the direct mixing of shielding gases for coated electrode arc welding (SAEE or SMAW), but also for the generation of standard gases.

These latter cases are useful in many applications, since they can be stored in cylinders and reused for other processes [19,20] or can be directly consumed online in the case of the calibration of an analytical device [21,22,23]. In addition, analytical instruments with low detection limits have been in much demand for many decades. Thus, they require accurate and reliable calibrations. For example, liquid standards are used for the calibration of spectrophotometers [24,25] and High Performance Liquid Chromatography (HPLC) devices. Standard gases are mainly used for the calibration of sensors [26,27,28], gas chromatographs [29,30] and other fluorescence detectors [31]. This calibration, on which the reliability of the measurements made by the analytical apparatus strongly depends, is crucial and must be accurate, repeatable, and reproducible in order to guarantee the quality of measurements over time.

The Institute of Chemistry and Processes for Energy, Environment and Health (ICPEES, Strasbourg, France) and In’Air Solutions have codeveloped a Benzene, Toluene, Ethylbenzene and Xylenes (BTEX) microanalyzer [32,33,34,35]. This analyzer has several major advantages: a detection limit of 1 ppb, a low sampling flow rate of 15–50 NmL min^−1^ and an increased portability for on-site measurements (6 kg and small footprint). This last advantage is important because it allows control over the indoor air quality in public buildings, whose pollution has the greatest impact on the population health.

Unfortunately, this key point is strongly impacted by the lack of calibration devices on the market that combine precision and portability. Indeed, most analyzers are currently calibrated in the laboratory, and are not recalibrated until they return. This is mainly due to the very high dilution rates, in the range of 1 to 10 L min^−1^, which are required to generate gas pollutant concentrations on the order of ppb, as shown in Table 1.

This leads, on the one hand, to the need for large and therefore cumbersome gas tanks, and on the other hand, to a considerable loss of compounds via a permanent leak when a lower flow rate is required for calibration.

Indeed, the most conventional method to produce pollutant concentrations ranging from 1 to 100 ppb requires the use of a gas cylinder with a concentration greater than or equal to 100 ppb, typically two commercial gas BTEX mixtures at either 100 or 1000 ppb of each compound. In this case, the method for diluting this stock gas mixture is to continuously mix a carrier gas flow rate (*Q_carrier_*) with a pollutant flow rate (*Q_pol_*) described in Figure S1a. The ratio $\frac{Q_{pol}}{Q_{carrier}+Q_{pol}}$ is then equal to the resulting dilution ratio, denoted herein as R. To achieve a concentration of 1 ppb with a carrier gas flow rate of 25 NmL min^−^^1^ and a mother gas mixture of 1000 ppb (i.e., R = 1000), it is necessary to be able to generate a BTEX gas flow rate of 25/1000 NmL min^−1^, i.e., 25 NµL min^−1^. This is just not possible with any actual gas flow regulators.

To overcome this problem, a novel gas mixture generator equipped with a micromixer, already numerically studied [36], and operating with a typical total gas flow rate of 25–50 NmL min^−1^, has been developed in this work as part of the European LIFE Smart In’Air project. The purpose of this new gas mixture generator was to combine precision and portability, in order to build on the advantages of the aforementioned BTEX microanalyzer. Thus, it should (i) allow calibration in the range required by the analyzer, i.e., between 1 and 100 ppb; (ii) generate these concentrations at a flowrate of c.a. 25–50 NmL min^−1^; and (iii) have a minimum size and weight (under 5 kg).

In order to achieve a continuous and very low pollutant flow rate, the gas flow can be divided into discrete pulses as illustrated in Figure S1b. Carrying out short pollutant gas trains interspersed with pure carrier gas trains allows us to achieve a dilution ratio that now depends on the ratio between the durations of the gas trains. This results in a so-called “temporal dilution”, with a temporal ratio (*R_temp_*) defined as follows:

(1) $$R_{temp}=\frac{t_{pol}}{t_{pol}+t_{carrier}}$$

where *t_pol_* is the duration of a pollutant train and *t_carrier_* that of a pure carrier gas train. For example, setting such *t_pol_* at 1 s and *t_carrier_* at 9 s would allow a dilution by one decade. To help in getting a constant pollutant concentration over time, a novel micromixer was designed, fabricated and placed downstream of the gas mixture generator.

Such a configuration has been numerically studied to investigate its potential mixing capability [36]. By means of computational fluid dynamics, our research group has already demonstrated that a homogeneous gas mixture could be obtained at the outlet of the micromixer.

The present work aims at experimentally validating the above modular configuration, which makes it possible to generate gas mixtures with extremely low final concentrations (1 to 100 ppb), while using low flow rates typically varying between 25 and 100 NmL min^−1^, unlike conventional apparatuses. Indeed, the latter usually work with a total gas flow rate of 1 to several NL min^−1^ to perform high dilution. This novel method used for the generation of gas pulses is described in detail below as well as in the calculations of the gas mixture concentrations generated and the experimental development and fabrication of a gas micromixer. The experimental validation is presented in the results section.

## 2. Materials and Methods

### 2.1. Method Used to Generate Gas Pulses

Herein, the method used to create pulses consists of a solenoid valve that alternately generates pure and polluted carrier gas trains. Such an operating procedure was described and investigated by Martin et al. [37] In their system, the gaseous pollutant mixture was generated through a permeation tube, a means of producing low flow rates of pure pollutant by evaporation, but which depends on precise control of parameters such as temperature [38]. However, the use of a commercial gas cylinder at a fixed concentration as a source of gas pollutants could alleviate the need to precisely regulated the temperature of the permeation membrane tube. In addition, the apparatus presented by Martin et al. showed a permanent outlet leak of the carrier gas when a pollutant was generated, and vice versa, resulting in a significant gas loss.

One method to eliminate this disadvantage is the one used for sample injection in gas or liquid chromatography [39,40]. This process involves a 6-way 2 position solenoid valve. The first position allows it to fully fill the sampling loop with the desired sample, while the gas or liquid mobile phase is injected into the chromatographic column and then into the detector for separation and detection, respectively. When the solenoid valve position is switched, the content of this loop is carried away by the mobile phase to the chromatography column, thus allowing the injection of a small sample volume into the mobile phase.

**Figure 1:**
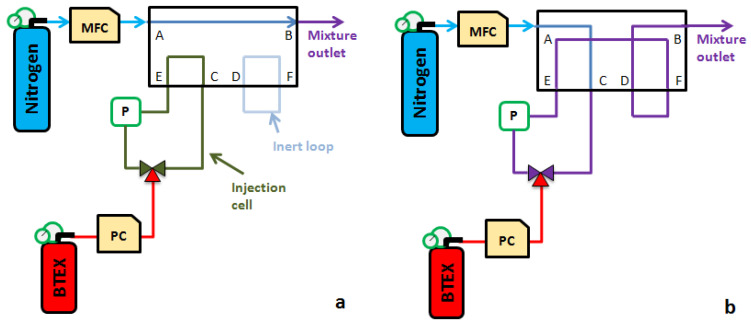
Schematic diagram of the gas pulse generator with a 6-way 2 position solenoid valve allowing it to generate a pure nitrogen gas train (**a**) and a nitrogen train loaded with a gas pollutant (**b**).

This analogy drove us to build a dilution device from a 6-way 2 position solenoid valve (MTV-6SL-N32UF-1, Takasago Fluidic Systems, Nagoya, Japan) as shown in Figure 1. Indeed, it is possible to create the pulses of a pollutant in a loop isolated from the continuous flow of carrier gas and then purge its content by changing the position of the solenoid valve. However, in the operation presented for liquid chromatography, the sample circulates permanently and thus requires a leak. To avoid this disadvantage, it is possible to transform the open sampling loop into a closed one. This closed loop is called hereafter “injection cell”. The injection of a pollutant into this cell is then performed thanks to a 3-way solenoid valve at 2 positions, where 2 ways are part of the cell, and the third way is connected to a pressurized gas pollutant cylinder of a fixed concentration (see Figure 1).

### 2.2. Calculation of the Generated Gas Mixture Concentration

The injection cell is initially filled with a pure carrier gas at near-atmospheric pressure. When the 3-way solenoid valve is in position 1, its third channel, in red in Figure 1a, is directly connected to the injection cell. Thus, due to the pressure difference between the tube located downstream to the Pressure Controller (PC in Figure 1) and the injection cell itself, the gas pollutant enters the injection cell, inducing a pressure increase. In this way, a known partial pressure of gas pollutant is added to the quasi-atmospheric pressure of pure carrier gas initially present in the injection cell. This allows to carry out a first dilution of the base mixture, called “dilution by injection”, whose *R_inj_* ratio is given by:

(2) $$R_{inj}=\frac{P_{pol}}{P_{atm}+P_{pol}}=\frac{P_{pol}}{P_{cell}}$$

where *P_atm_* and *P_pol_* are the atmospheric pressures of the carrier gas already present in the injection cell and the partial pressure of the gas pollutant added into the injection cell, respectively. *P_cell_* is the final pressure reached once the pollutant has been added.

It is then possible to vary the value of *R_inj_* by changing the partial pressure *P_pol_* thanks to the pressure controller (IQ-Flow, Bronkhorst, Montigny-lès-Cormeilles, France) (see Figure 1). Indeed, the downstream pressure imposed by the regulator gives the final value of the pressure in the injection cell. Once this pressure is reached, the 3-way solenoid valve moves to position 2, thus isolating the injection cell whose content is ready to be purged. To determine if the final pressure in the cell is the expected one, an online pressure sensor (MPS3, Elveflow, Paris, France) was used to monitor the pressure variation.

The cell is then purged in the same way as for the sample injection into the chromatography column. The 6-way solenoid valve changes position, thus redirecting the carrier gas flow towards the injection cell whose content is flushed. This forms a carrier gas train loaded with a single gas pollutant pulse. The purging time depends on 3 parameters: (i) the carrier gas flow rate *Q_carrier_*; (ii) the total pressure of the injection cell *P_cell_*; and (iii) its volume *V_cell_*. Thus, for the cell to be fully flushed, the flush duration *t_flush_* must be longer than:

(3) $$t_{flush}=\frac{V_{cell}}{Q}\times \frac{P_{cell}}{P_{\mathrm{atm}}}$$

In this study, the cell volume was defined based on the following parameters:

*Q_carrier_* = 25 NmL min^−1^, the minimum flow rate for the mixing generator;

*P_cell_* = 2000 mbar, the maximum total pressure of the cell corresponding to a two-fold dilution (1000 mbar of pure carrier gas and 1000 mbar of pollutant);

*t_flush_* = 1 s, the fixed purge time of the cell.

Thus, the necessary cell volume was calculated to be equal to *V_cell_* = 208.33 µL.

Once the content of the cell has been purged, the 6-way solenoid valve changes position again, allowing pure carrier gas to be generated during a new filling of the cell. Alternating pure carrier gas trains and pollutant-loaded carrier gas trains then make it possible to achieve a second dilution called “time dilution”. The ratio between the pulse duration of a pure carrier gas train and the pulse duration of a carrier gas train loaded with pollutant gives the ratio of this second dilution as established in Equation (1).

By combining the dilution by injection (Equation (2)) and the temporal dilution (Equation (1)), the final dilution ratio *R_final_* of the base mixture is obtained as follows:

(4) $$R_{final}=R_{temp}\times R_{inj}=\frac{t_{pol}}{t_{pol}\times t_{carrier}}\times \frac{P_{pol}}{P_{cell}}$$

**Figure 2:**
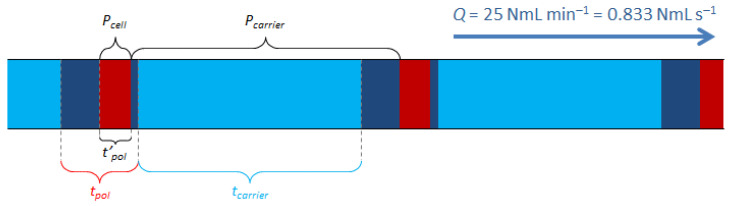
Schematic drawing of the flow composition and pressure exiting the mixing generator. The usual total flow rate is Q = 25 NmL min^−1^.

However, the use of a 6-way solenoid valve for this operation implies the presence of a so-called “inert” loop in the circuit, as shown in Figure 1, which contains a given volume of carrier gas at quasi-atmospheric pressure, in addition to the final dilution ratio (Equation (4)). Considering the dilution by injection, the dilution time and the content of this inert loop, we can see that the flow is composed of many gas trains whose duration and pressure are important in the calculation of the final concentration (Figure 2).

Finally, the fixed duration of 1 s of a carrier gas train loaded with pollutant implies, in cases where *P_cell_* is less than 2000 mbar, an excess purging of the injection cell content. It is then necessary to consider the excess of pure carrier gas generated during this duration, i.e., 1 s. This additional dilution is once again composed of pure carrier gas at near atmospheric pressure. This leads to no longer considering the duration of a polluted carrier gas train with the value *t_pol_*, but with a new lower value *t’_pol_* as illustrated in Figure 2. *t’_pol_* is defined according to the following Equation:

(5) $$t_{pol}^{\prime }=\frac{V_{cell}}{Q}$$

It is thus necessary to correct *t_pol_* in Equation (4) by replacing it with *t’_pol_*.

In addition, it is also necessary to consider the overpressure of the cell content. Indeed, a *P_cell_*/*P_carrier_* factor must be added to the dilution ratio to complete the equation. Finally, we obtain a flow composed of 4 distinct segments, whose characteristics are given in Figure 2 according to a schematic representation of the cycles. Taking all these data into account, the corrected final dilution ratio *R’_final_* can be determined according to the following Equation:

(6) $$R{{}^{\prime }}_{final}=\frac{t{{}^{\prime }}_{pol}}{t_{pol}\times t_{carrier}}\times \frac{P_{pol}}{P_{cell}}\times \frac{P_{cell}}{P_{carrier}}=\frac{t{{}^{\prime }}_{pol}}{t_{pol}\times t_{carrier}}\times \frac{P_{pol}}{P_{carrier}}$$

### 2.3. Chemicals

The BTEX concentrations were generated from two different standard BTEX mixtures purchased from Messer (Folschviller, France) with an initial concentration of overall compounds equal to either 100 ppb or 1000 ppb and with a 10% uncertainty for both gas cylinders. Both standard mixtures were diluted with pure air or pure nitrogen (99.999% purity, Messer, Folschviller, France) to obtain the targeted concentrations of BTEX mixtures.

### 2.4. Measuring the Generated Gas Mixture Concentrations

The apparatus used for the analysis of the concentration produced by the generator of mixtures is a BTEX analyzer (Benzene, Toluene, Ethylbenzene and Xylenes) provided by the company In’Air Solutions (µBTEX-1, Strasbourg, France). This device is a portable and compact Gas Chromatograph (GC), which allows these compounds to be separated to measure their gas phase concentrations.

The working principle of this analyzer, developed by Nasreddine et al. in 2015 [34], has been widely described in recent publications [33,35]. Analyzing a sample consists of 3 steps: sampling, separation, and detection. During sampling (Figure S2a), a pump (SP570.ECBLa, Schwarzer Precision GmbH, Essen, Germany) makes the sample to flow through a 6-way/2 positions valve and a sampling loop of 200 µL. The flow rate of the sample is limited by an external Mass Flow Controller (MFC) (IQ-Flow, Bronkhorst, France) placed upstream to the pump.

The sample passes continuously through the 200 µL-loop. In parallel to this sampling, a flow of pure nitrogen, used as carrier gas, flows through a capillary column and then through a Photo-Ionization Detector (PID) (piD-TECH eVx blue, Baseline, Baseline-MOCON Inc., Lyons, GA, USA) at a flow rate of 2.5 NmL min^−1^, this flow rate being regulated by a Pressure Controller (PC) (IQ-Flow, Bronkhorst, Montigny-lès-Cormeilles, France) placed upstream to the 6-way valve.

The second step, consisting of separating the compounds of the sample, starts by switching the 6-way valve (Figure S2b). The gaseous sample inside the 200 µL-loop is then flushed by the carrier gas and injected into the capillary column. Here, the compounds are separated depending on their affinity with the solid phase of the chromatographic column (internal diameter (ID) 0.18 mm, RXi-624 stationary phase, 1 µm film thickness, Restek, Bellefonte, PA, USA), which differs according to each compound’s physical properties. Indeed, Benzene is less retained by the column while o-Xylene is longer adsorbed on the capillary column walls.

After exiting the column, each compound passes through the photoionisation detector (PID) where it is quantified for the last step by means of an external calibration performed prior the experiments. For BTEX, compounds exit the capillary column in this order from first to last: Benzene, Toluene, Ethylbenzene, m- and p-Xylenes (co-eluted) and o-Xylene. The signal provided by the PID is then obtained in µV: the more molecules detected, the higher is the signal. The concentration of a compound can then be determined by the area under its related signal peak.

### 2.5. Experimental Development and Fabrication of a Gas Micromixer

In order to ensure a fast response time and the highest possible homogeneity to the gas flow leaving the above gas mixture generator, a microfluidic homogenization device had to be developed [36]. So far, axial gas heterogeneities have been poorly represented and investigated. Martin et al. (2012) proposed a homogenization method, but it was limited because it was specifically designed for their application and a given time dilution ratio, which did not match with our need for a flexible homogenization method. However, in the case of the gas mixture generator presented here, achieving the desired concentration range requires the ability to modify this dilution ratio.

Thus, a microfluidic homogenization circuit was investigated. The experimental development reported hereafter was supported by a previous theoretical study conducted by Computational Fluid Dynamics (CFD) [36]. In the case of gas mixtures, it should be noted that the diffusion coefficient D between gases (0.1 cm^2^ s^−1^) is significantly higher than that between liquids (1.10^−5^ cm^2^ s^−1^), i.e., typically four orders of magnitude higher. In fact, it becomes very interesting to take advantage of this natural phenomenon for mixing gas trains. To achieve this, it is necessary to reduce the value of the Peclet number (Pe) for the gas stream studied. Indeed, this number represents the ratio of a mass transport rate by convection and by diffusion:

(7) $$Pe=\frac{L_{c}\times v}{D}$$

where *L_c_* is a characteristic length (cm), *v* is the local gas velocity (cm s^−1^) and *D* is the mass diffusion coefficient (cm^2^ s^−1^).

In order to give more importance to mass transport by diffusion, it becomes necessary to reduce the convection term, and thus to reduce the average velocity *v* of the fluid. The concept of a buffer tank allows us to achieve this objective by increasing the cross-section through which the gas mixture flows. Thus, the larger the cross-section of the buffer tank compared to the upstream channel, the more homogenous the mixture will be.

To ensure an efficient mixing at a flow rate of 5 NmL min^−1^, a buffer tank was considered to mix a pure 9 s carrier gas train with a 1 s pollutant-loaded carrier gas train, i.e., a volume of 0.833 mL. These conditions correspond to the extreme case of the “temporal dilution” in the present work. Consequently, it was arbitrarily chosen to build a tank to hold 1.5 times this volume, i.e., about 1.2 mL. This first tank had the dimensions 30 × 40 × 1 mm (length × width × depth).

**Figure 3:**
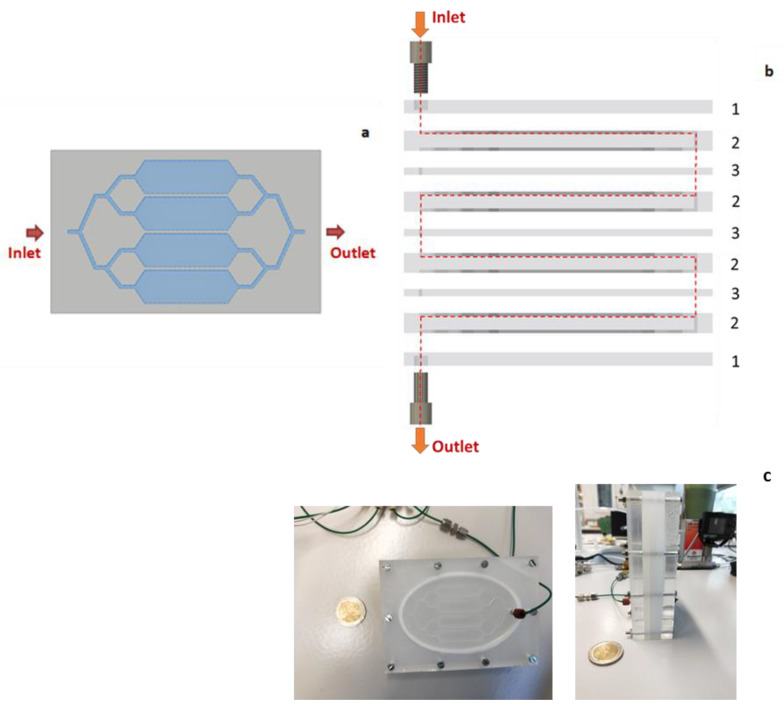
Schematic drawing of (**a**) the design of a 4 buffer stage as numerically studied by Noel et al. (2019) [36] for which dimensions can be found in Figure S3A; (**b**) image of a 4-stage micromixer [36] comprising four mixing plates milled on one side with a four buffer tanks (2), one inlet and outlet plate (1) as well as one connection plate (3) to link two mixing plates; (**c**) photographs of the 4-stage micromixer prototype manufactured and tested in this work.

However, a section too large may involve dead zones whose volumes are not renewed (i.e., flushed), thus impacting the response time of the overall circuit. To avoid this phenomenon as much as possible, it was chosen to rely on 4 smaller buffer tanks placed in parallel. It thus offers the same overall cross-section increase while limiting possible dead zones and offering an additional benefit arising from the 4-fold reduction of the convective mass transport. We then obtain a pattern of 4 tanks, each with dimensions of 30 × 10 × 1 mm (Figure 3a and Figure S3A).

Finally, in order to meet the need for homogenizing mixtures generated at flow rates equal or above 25 NmL min^−1^, we considered modular gas micromixers composed of the assembly of X-alternated rectangular buffer stages (Figure 3a) and connection stages, X being the number of buffer stages, i.e., the number of mixing plates as defined in Figure 3. Figure 3b represents the assembly of a 4-stage micromixer needed to homogenize the axial heterogenous gas mixture at a flow rate of 25 NmL min^−1^. Each buffer stage (79 × 112 × 10 mm) comprises 1 pattern of 4 buffer tanks with their inlet and outlet network of microchannels (1300 × 1300 µm) (Figure 3b). Connection stages of a lower thickness (5 mm) comprise transversal microfluidic channels (1300 × 1300 µm) for connecting two buffer stages or to fix a 1/16” fluid connector. Thus, the entire assembly represents a multi-stage micromixer.

A laboratory prototype of this micromixer was developed and tested as part of this study (Figure 3c). It was made of Poly(methyl methacrylate) (PMMA), a material inert to the pollutants studied, and the patterns were engraved using a micro-milling machine (Roland MDX-40A, Serris, France). The connectors used for the mixer inlet and outlet tubes were 1/16” Polyetheretherketone (PEEK) connectors. Sealing between the stages was ensured by 3 mm diameter Teflon O-rings, inserted into grooves, and compressed with screws passing through the entire mixer.

In order to manufacture the stages of the micromixer faster, two different milling tools were used (Figure S3B). At first, the raw shape of the 4 buffer zones pattern, the screw paths and O-ring housing, were conducted with a 1.5 mm milling tool (green on Figure S3B). Once this step was achieved, the microchannels were manufactured using a 0.5 mm milling tool (red on Figure S3B), leading to satisfactory precision.

## 3. Results

**Figure 4:**
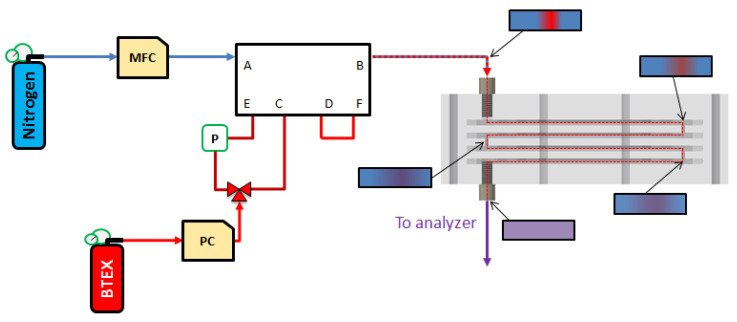
Illustration of the setup after adding a 4-stage micromixer to the original gas mixture generator. The inlet and outlet plates (plates 1 in Figure 3b) as well as the connection plates (plates 3 in Figure 3b) to link the mixing plates are not displayed for clarity. Insets present the evolution of the pollutant heterogeneity through the micromixer as determined by a previous simulation work [39], red being the polluted sample, blue the pure carrier gas and purple the perfect mixture of the two.

The objective of this work is to compare the experimental results obtained with the gas mixture generator coupled or not to the gas micromixer described above and check the consistency with the results determined numerically by Computational Fluid Dynamics (CFD) in a previous work [36]. The purpose of adding a micromixer was to ensure a rapid homogenization of the gas train generated as illustrated in Figure 4. CFD simulations carried out in a previous study [36] highlighted a homogenization capacity of more than 95% for flow rates between 25 and 100 NmL min^−1^. These performances were achieved with a 4-stage micromixer operating at a flow rate of 25 NmL min^−1^, 8 stages for 50 NmL min^−1^ and 16 stages for 100 NmL min^−1^. These numerical results indicated a linear trend between the number of stages needed, and the gas flow rate applied.

Consequently, the present experimental work was limited to the realization and study of a mixture gas generator equipped with a 4-stage micromixer operating at 25 NmL min^−1^.

### 3.1. Gas Mixture Generator Alone or with a Dowstream Micromixer

First, experiments were performed using the gas mixture generator alone. A representative gas mixture at 50 ppb of each BTEX was thus chosen and generated from a commercial gas cylinder of 1000 ppb of each compound, at a flow rate of 25 NmL min^−1^. This targeted concentration of 50 ppb was reached with *P_pol_* = 200 mbar and *t_carrier_* = 1 s, i.e., an estimated final concentration of $C_{final}=C_{1000ppb}\times R{{}^{\prime }}_{final}=49.92\;\mathrm{ppb}$, where *C*_1000*ppb*_ is the concentration of the commercial BTEX gas cylinder.

**Figure 5:**
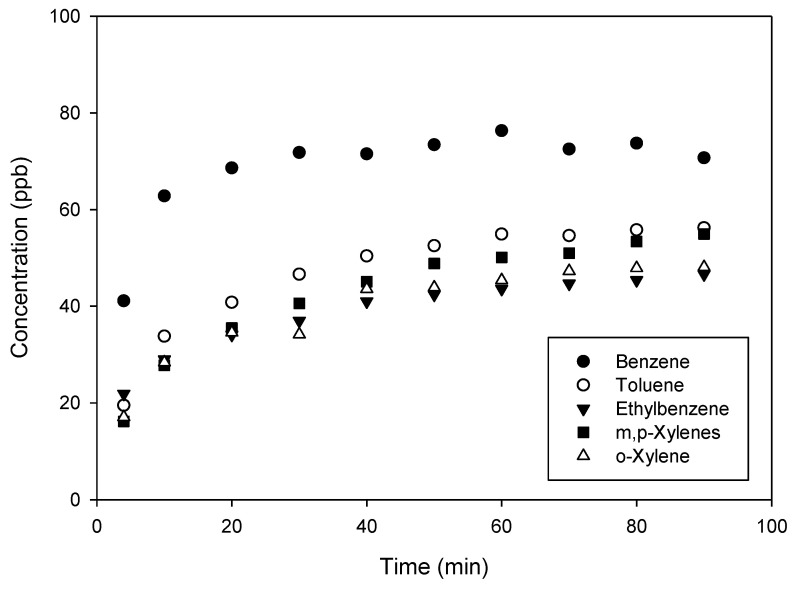
BTEX concentration time profiles were obtained without any micromixer by generating a targeted gas mixture of 50 ppb from a gas cylinder of BTEX at 1000 ppb of each compound. Other parameters were fixed at: Q = 25 NmL min^−1^; *P_pol_* = 200 mbar; *t_carrier_* = 1 s. For m,p-Xylenes, the concentration reported corresponds to the individual concentration of each compound.

As shown in Figure 5, the response time of the generation device was quite long, in the order of several tens of minutes. Indeed, the concentration of each compound stabilized after approximately 60 min and was, however, very close to the target value of 50 ppb, which constituted a first proof of concept. However, the concentrations were all slightly below 50 ppb, which was explained by the fact that the cell pressure *P_cell_* did not reach the desired value: a smaller amount of pollutant was then introduced during the dilution by injection.

One also noticed that the final benzene concentration of around 70 ppb was significantly higher than the expected 50 ppb. This was explained by a defect in the calibration of this compound, which was subsequently corrected. Despite this fact, benzene had a response time close to that of other compounds, and an equivalent stability once the steady-state regime had been established.

To solve this issue related to the response time, the optimization of the tube lengths was carried out in the whole experimental device before carrying out more tests. The aim of this optimization was to drastically reduce the total volume of the circuit and to limit dead volumes as much as possible. The total volume was thus divided by approximately 10, from more than 80 mL down to 7.4 mL.

In addition, the analysis method was modified to obtain closer measurement points over time. Thus, the chromatography column was removed to let the mixture fed directly the PID detector, thus offering a measurement point every 2 min instead of every 10 min. In fact, the separation of the compounds required approximately ten minutes, which determined the time resolution, i.e., the time between two analyses as soon as the chromatographic column was present.

In the absence of the column, the compounds were no longer separated, which made it possible to determine an overall concentration of the BTEX injected by considering that the response factor of these 6 species was similar, as reported in the literature [41]: 0.780 (Benzene); 0.794 (Toluene); 0.818 (Ethylbenzene); 0.812 (m, p-Xylenes); 0.840 (o-Xylene). This assumption was verified experimentally later since the total concentration generated was close to the targeted one.

**Figure 6:**
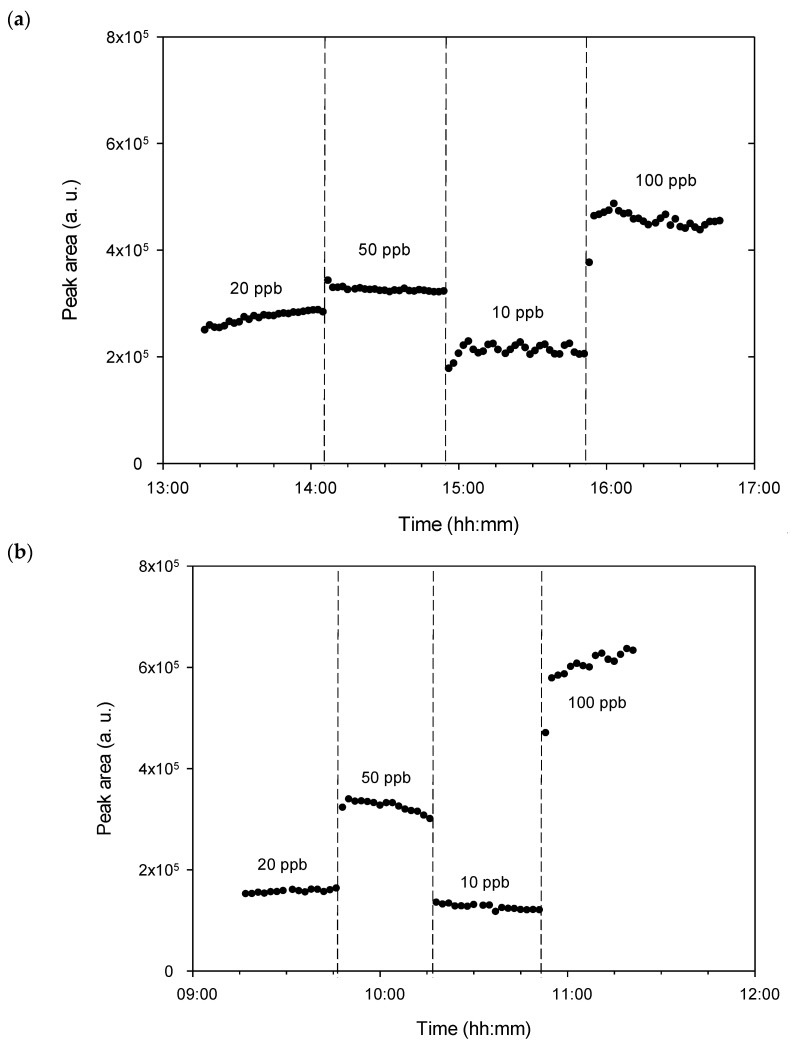
Total BTEX peak area on a logarithmic scale vs. time obtained either (**a**) without any micromixer or (**b**) with the gas train generator coupled to a 4-stage micromixer for gas mixtures of 20, 50, 10 and 100 ppb generated from a gas cylinder of BTEX at 1000 ppb of each compound. Concentrations of 20, 50, 10 and 100 ppb were generated with respectively *P_pol_* = 200 mbar and *t_carrier_* = 4 s, *P_pol_* = 200 mbar and *t_carrier_* = 1 s, *P_pol_* = 200 mbar and *t_carrier_* = 9 s, and *P_pol_* = 400 mbar and *t_carrier_* = 1 s. The total flow rate was fixed at *Q* = 25 NmL min^−1^.

New tests were then performed with the new configuration with a lower dead volume, generating BTEX mixtures at 20, 50, 10, and 100 ppb, consecutively (Figure 6a). A significant improvement in the response time was then observed, since the latter was reduced down to 4 min. On the other hand, the stability of the concentration was not ensured, the concentration of 10 ppb presenting a significant variation in concentration over time. The maximum and minimum areas obtained for this concentration of 10 ppb from each of the BTEX were equal to 2.29 × 10^5^ a.u. and 2.05 × 10^5^ a.u., respectively, which represented a variation of about 10%.

Therefore, even if the response time was considerably improved thanks to the reduction in the volume of the circuit, the instability of the concentration nevertheless required the use of a micromixer placed after the gas train generation in order to reduce the BTEX concentration fluctuation over time. This periodic instability (see the lowest concentration of 10 ppb in Figure 6a) also confirmed the axial heterogeneity already observed in our previous simulation work (Noel et al., 2019) [36]. The fact that these periodic variations did not appear for the concentrations other than 10 ppb came from the highest temporal dilution of 1/10 used in this case (*t_pol_* = 1 s, *t_carrier_* = 9 s), while other concentrations were produced with the smaller temporal dilution of 1/2 or 1/5, implying less axial heterogeneity.

In order to confirm the numerical results, the same tests were carried out experimentally at 25 NmL min^−1^ by generating BTEX mixtures of 20, 50, 10, and 100 ppb from a BTEX gas cylinder at 1000 ppb, and by measuring the total concentration of BTEX generated at the outlet of the micromixer. The results are presented in Figure 6b and show a further significant improvement in the response time, which now appears to be less than the 2 min required for the measurement. In addition, the variations in concentration previously observed for 10 ppb due to poor homogenization in the absence of the micromixer have completely disappeared, with no periodic variations in the concentration at the micromixer outlet being visible.

Furthermore, in the presence of the micromixer (Figure 6b), it was not possible to observe the oscillations predicted by the simulation (Figure 6 shown in our numerical paper [36]) because the time step of the measuring instrument was 2 min, whereas the period of the oscillations observed numerically was only about 10 s.

### 3.2. Calibration and Reproducibility Experiments

**Figure 7:**
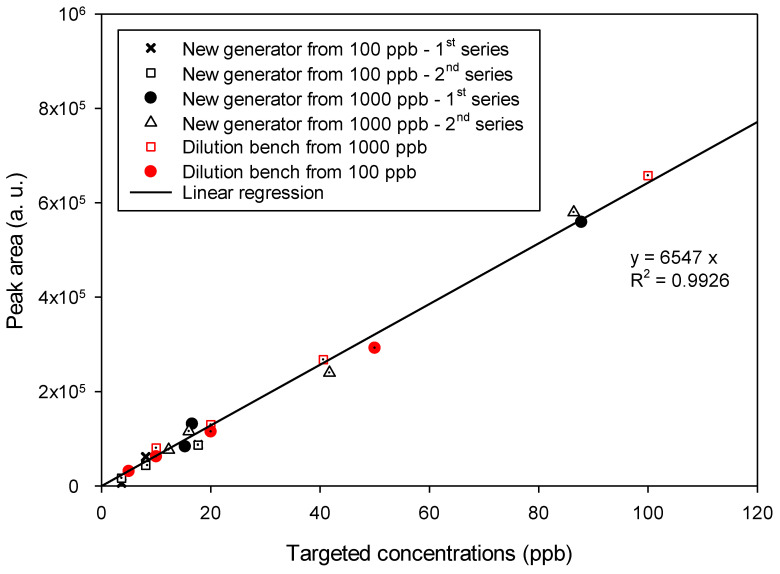
Comparison of the results obtained with the dilution bench and the novel source of gas mixtures based on a gas train generator coupled to a 4-stage micromixer, both using a gas cylinder of BTEX at 100 or 1000 ppb of each compound. The plot represents the experimental chromatographic peak area vs. the targeted concentration. The red symbols correspond to the data obtained with the dilution bench while the black ones were obtained with the experimental generator equipped with a 4-stage micromixer. The flow rate was fixed at: *Q* = 25 NmL min^−1^. *P_pol_* varied from 100 to 2000 mbar and *t_carrier_* varied from 1 to 9 s.

The reproducibility of the novel gas mixture generator was then verified by overlapping several series of measurements carried out under different conditions and spaced over time (see Figure 7). Similar measurements performed with a laboratory validated calibration bench were also added to compare the data obtained with the new microdevice with a reference generation source so-called dilution bench.

For each series of measurements, areas of the peaks obtained under conditions for the generation of variable BTEX mixtures were corrected by subtracting the average value of the peak obtained with pure nitrogen for 1 h after stabilization. This theoretically allows us to obtain a straight line passing through the origin when plotting the variations of areas as a function of pollutant concentration. These concentrations were recalculated from the exact experimental pressure data recorded using the pressure sensor.

The concentrations targeted and obtained are listed in Table S1 for the different series of experiments performed at *Q* = 25 NmL min^−1^, combining both generation and measurements.

### 3.3. Experiments at Different Total Flow Rates

A last experiment was carried out to compare the gas mixture generation of the same concentration at different flow rates. Indeed, the generator of gas mixtures needs also to be able to operate at different flow rates to allow the calibration of a large range of analytical instruments such gas analyzers and gas sensors. Thus, flow rates of 12.5, 25 and 50 NmL min^−1^ were investigated to obtain a fixed target BTEX concentration of 50 ppb (Figure S4).

## 4. Discussion

### 4.1. Gas Mixture Generator with Dowstream Micromixer

A thorough analysis of Figure 6b shows that, for every given concentration generated, the obtained signal slightly increased or decreased depending on the concentration considered. This is because the total pressure in the cell varies around the target pressure *P_cell_*, indeed the pressure regulator used did not close completely when the pressure of its setpoint was reached. Therefore, and since the pressure upstream to the regulator was always higher than the set pressure, it continued to allow the BTEX mixture to pass through it, the pressure downstream then varying regularly.

An accurate control of the pressure would make it possible to a achieve better precision and stability of the concentration generated. To fix this problem, an assembly coupling a proportional solenoid valve to a pressure sensor could be considered in the future, since this type of solenoid valve can be completely closed to stop the arrival of a pollutant train when the desired pressure *P_cell_* is reached.

However, this concentration variation, due to the pressure management, was nevertheless corrected post-generation by calculating the true concentration generated. Indeed, the software developed for the control of the gas mixture generator allowed us to record and reprocess the data from the pressure sensor. It was thus possible to know at each generation cycle the real pressure introduced into the cell (*P_cell_*) and therefore to calculate the exact concentration generated using Equation (6).

Even if the concentration varied compared to that of the set value entered in the software controlling the gas mixture generator, it was possible to know the final concentration generated. The accuracy of the pressure sensor being 0.2% of full scale and its maximum pressure being 7 relative bars, it was possible to detect pressure changes in the order of 14 mbar. This was equivalent to a potential error of +/− 7% for a pressure *P_pol_* = 200 mbar used to obtain a gas mixture containing 50 ppb of each compound.

This error could be reduced by selecting another sensor from the same series available from the same manufacturer, such as an MPS3 pressure sensor, whose maximum relative pressure is 2 bars. Indeed, the mixture generator should not exceed a cell pressure *P_cell_* of 2000 mbar absolute. The accuracy of this sensor also being 0.2% of full scale, the potential error would be reduced down to +/− 2%, always for a pressure *P_pol_* = 200 mbar.

Concerning this concentration variation phenomenon, it also appeared in another experiment, during which a zero concentration (pure nitrogen) and a concentration of 50 ppb of each BTEX were alternately generated. Figure S5 shows that the repeatability of the concentration generated is very satisfactory. Indeed, Figure S5 exhibits that the two generation periods of 50 ppb are extremely similar to each other, both in terms of the final value reached and in the shape of the curve.

For the four targeted concentrations of 20, 50, 10 and 100 ppb of BTEX (Figure 6b), the mean peak areas were respectively 15.7 × 10^4^ a.u., 32.5 × 10^4^ a.u., 12.6 × 10^4^ a.u. and 60.9 × 10^4^ a.u. These mean values were respectively linked to standard deviations of 0.3 × 10^4^ a.u., 1.1 × 10^4^ a.u., 0.5 × 10^4^ a.u. and 1.8 × 10^4^ a.u. The calculated standard deviations represent thus between 2.1% and 4.1% of deviation around the mean value, depending on the generated concentration.

According to Figure 6b, a slight increase of the PID signal was observed for the concentration of 50 ppb after reaching the equilibrium regime. After stabilization, the variations between the lowest and highest value of a 50 ppb BTEX generation series for 1 h were in the order of 10%. However, due to the pressure variation at the injection cell level, this error could be corrected by recalculating it using the recorded data from the pressure sensor to know the exact concentration generated during each cycle.

In Figure S5, it could be also observed that several tens of minutes were necessary for the PID signal to stabilize when pure nitrogen was injected alone. This continuous flow of pure nitrogen was injected by the same path as the pure nitrogen pulses produced when generating a polluted mixture. For this purpose, the 6-way valve did not switch during pure nitrogen generation. It also ensured that the flow of pure nitrogen was isolated from the BTEX injection valve. This was an additional security measure to prevent unwanted BTEX amounts from getting through the valve and polluting the flow of pure nitrogen.

Since the response time of the gas mixture generator coupled with the gas micromixer was less than 2 min when switching from one concentration to another, as seen previously in Figure 6b, the memory effect due to potential adsorption on the PEEK device and Teflon tubes did not seem to be an issue. However, the renewal time of the PID detector cell could support, at least partially, these observations; the BTEX molecules being able to adsorb onto the surface of the porous membrane located at the detector inlet.

Being exposed to pure nitrogen, the peak area of the PID detector stabilizes at around 6 × 10^4^ a.u in Figure S5. This blank value defining the baseline, when the BTEX analyzer was configured as a chromatograph equipped with a GC column, may vary slightly between two series of measurements spaced over time (several days or weeks for example).

### 4.2. Calibration and Reproducibility Experiments

Figure 7 groups together all the results obtained from the different series of measurements made with two different BTEX cylinders at 100 or 1000 ppb respectively combined with the dilution bench or the new generation source equipped with the micromixer. The peak area of total BTEX increased perfectly linearly with the concentration of BTEX in the range 3.7–100 ppb, the linear regression of the dataset allowed to obtain a straight line with a coefficient of determination R^2^ = 0.993. In addition, the data obtained with the new generation source agreed perfectly with those obtained by the reference method, namely the dilution bench.

This confirms the ability of the new microfluidic gas mixture generator to produce a reliable linear calibration plot in a concentration range of 5 to 100 ppb, with initial standard BTEX gas mixtures of different concentrations. In addition, the fact that the measurements were taken between several days and 2 weeks apart also made it possible to validate the reproducibility of the gas mixture generation thanks to the new microdevice.

The standard deviation on the peak area was equal to about 1.70 × 10^4^ a.u., which indicates an uncertainty greater than 10% for concentrations less than or equal to 20 ppb. Indeed, the area obtained from the PID signal is less than 130,000 units for this concentration. Therefore, a calibration curve performed at low concentrations should contain many calibration points to ensure a reliable linear plot.

It is not certain, however, that this uncertainty was only due to the gas mixture generator. It is possible that the PID signal also included an error in the measurement, but also that the reprocessing of the data and particularly the integration of the peak, implied another error adding to measurement and generation of uncertainties. In this case, however, it was not possible to determine the impact of each contribution on the final error.

### 4.3. Experiments at Different Total Flow Rates

The results presented in Figure S4 show that the PID signal was stable for each flow rate investigated, indicating that this parameter did not influence the homogeneity and the stability of the concentration generated. On the other hand, a difference in PID signal was observed for the different flow rates. For a flow rate of 50 NmL min^−1^, the average value obtained was around 207,000 a.u., compared to 195,000 a.u. for 25 NmL min^−1^ and 148,000 a.u. for 12.5 NmL min^−1^.

The significant difference between the above value for a flow rate of 12.5 NmL min^−1^ and those obtained with the highest flow rates is most likely due to this low total gas flow rate, which no longer allowed the complete purging of the injection cell. Indeed, the volume of this latter has been sized to allow a total purge at 25 NmL min^−1^, which means that at a flow rate of 12.5 NmL min^−1^, only half of the 208 µL cell was purged. As a result, only half of the BTEX contained in the injection cell was flushed during the purge step, theoretically dividing the final concentration by 2.

The small difference between the signal obtained at 25 and 50 NmL min^−1^ in Figure S4 is probably due again to the poor management of the cell pressure (*P_cell_*). Indeed, the generation of an identical concentration at a flow rate of 25 NmL min^−1^ and at a flow rate of 50 NmL min^−1^ was obviously done by doubling the *P_cell_* value. However, a different cell pressure implies that the pressure regulator should be capable of managing the exact value of the pressure downstream.

The pressure regulation problems are illustrated in Figure S6. Here, two series of data are recorded using the cell pressure sensor during the generation of 50 ppb of BTEX with *P_pol_* = 200 mbar, *t_carrier_* = 1 s and a flow rate of 25 NmL min^−1^. The value of the relative pressure *P_atm_* must be a quasi-atmospheric pressure. This is relatively constant, and its average value over the entire generation time can be compared to the relative pressure *P_cell_* value to determine the pressure *P_pol_* of each cycle, allowing the calculation of the exact gaseous mixture concentration generated.

According to Figure S6, the relative pressure *P_atm_* was quite stable, with an average of 97.5 mbar and a standard deviation of 7.34 mbar, which corresponds to a variation of 7.5%. On the other hand, this figure highlights the problem of regulating the pressure *P_cell_* during the cycles. After a stabilization period where the cell pressure decreased from 550 mbar (setpoint from the previous generation experiment) to approximately 280 mbar (new setpoint), an oscillation of *P_cell_* was observed between 269 and 293 mbar with a period of about 34 s, leading to an average value of 281 ± 12 mbar, i.e., a variation of 4.3%.

This implies that at a flow rate of 25 NmL min^−1^, about 14.17 mL of the gas mixture flowed in a period of pressure oscillation. However, the BTEX analyzer (µ-BTEX1, In’Air Solutions) coupled to the generation source in this work had a sampling loop of only 200 µL. The cell pressure variation can thus strongly impact the detected concentration. Nevertheless, some of these variations were potentially damped by the micromixer during homogenization. Indeed, the volume of this device has been dimensioned to homogenize 10 s of mixture (*t_carrier_* = 9 s), with, in addition, an oversizing by a factor of 1.5. Therefore, in the future, this phenomenon should be investigated in detail in order to evaluate the impact on the final concentration, which could be measured by online mass spectrometry.

## 5. Conclusions

A new gas mixture generator was successfully conceived, designed, and manufactured to reach a dilution ratio up to 100 at a low flow rate of 25 NmL min^−1^. This total gas flow rate is about 40 to 200 times less than the operating flow rate of commercial calibration devices. Thus, this microfluidic generator allows generating gas concentrations as low as 10 ppb of a BTEX mixture from a 1000 ppb commercial cylinder. Its low carrier gas consumption eliminates the need for large, pressurized cylinders that limits the portability of the current equipment. For example, a 58 L-gas cylinder of nitrogen weighs only 1.2 kg and allows a continuous gas generation for 38.7 h when a full gas calibration of a gas analyzer with five known concentrations needs 5 times 10 min, i.e., a total time of 50 min. In addition, the on-site calibration of analytical instruments requires only 1 or 2 calibration points if no drift with the previous full calibration is observed. In addition, the low carrier gas consumption avoids the unnecessary loss of costly gas. The weight of the developed generator itself should not exceed 5 kg and was composed only of few solenoid valves, gas connectors and a gas micromixer placed downstream to the gas train generator.

The gas homogenization was achieved using a multi-stage microfluidic mixer and was higher than 95% for a response time of less than 2 min. The design of this new micromixer, which has been also validated by previous numerical study [36], allows homogenizing gas mixtures with axial heterogeneity, which was scarcely studied until now in the literature but seems to be of great potential in view of the results obtained during the present study. In addition, this micromixer has a modular design, which allows adding new mixing plates for increasing homogenization efficiency. These plates were manufactured by a simple etching process with a lab micro-milling machine but could be also manufactured by other microfluidic microfabrication techniques including 3D printing.

Finally, the performances of this gas mixture generator could be further improved by ensuring a better pressure regulation when BTEX mixture is injected into the injection cell of the generator. Indeed, the major source of errors on the concentration generated comes from a potential regulatory failure, which could easily be solved with another technology. For example, one could couple a pressure sensor and a proportional solenoid valve to replace the pressure regulator. Such an improvement could also further reduce the production cost and the low concentration limit of the gas mixture generator: it would then be possible to inject an even lower partial pressure of BTEX into the cell, which will further increase the dilution ratio per injection. As such, it would be possible to achieve a dilution ratio of up to 1000, and to reach concentrations of around 1 ppb of BTEX from a commercial cylinder of 1000 ppb.

In the future, to address the high cost of O-rings used in the assembly of the current micromixer, consideration will be given to fabricate the micromixers in one piece by 3D printing with BTEX non-emissive and non-adsorbing polymer materials. In addition, this type of gas generator could be used for gases other than BTEX, such as for other volatile organic compounds (alkanes, aldehydes, ketones, etc.) or even gaseous inorganic pollutants (oxides of nitrogen, sulfur compounds, carbon oxides, etc.) For example, this new tool could be used for calibrating NO and NO_2_ analyzers, or CO and NH_3_ sensors, etc.

## 6. Patents

The following patent resulting from the work reported in this manuscript has been submitted:

F. Noel, S. Le Calvé, C. Serra, Microfluidic generator for generating a gas mixture. WIPO Patent Application WO/2020/178022, 10 September 2020.

## Figures and Tables

**Table 1:** Limiting characteristics of the different mixture generating devices on the market.

	Type of Compounds	Weight (kg)	Range of Gas Concentrations (ppb)	Gas Flow Rate (NmL min^−1^)	Accuracy (%)
CGS-12	Gas	21		3000	1
HovaCAL^®^	Liquid and gas	15	1 to 2666 *	10,000	<2
FlexStream™	Liquid and gas	12.2	<1 to 10,000	10,000	- **
491Flex™	Liquid and gas	13.6	<1 to 10,000	10,000	- **
EcoFlex™	Liquid and gas	12.2	<1 to 10,000	5000	- **
Span Pac™ I	Liquid and gas	27.2	1 to 1000	5000	- **
KinTek 491M	Liquid and gas	14.5	1 to 1000	5000	- **
Dynacalibrator^®^	Liquid and gas	4.8	>10 to 1000	1200	- **
Span Chek™	Liquid and gas	5.2	1 to 100	1500	- **
OVG-4™	Liquid and gas	4.5	50 to 1000	11,000	- **

* For aqueous solutions containing 0.1% *w*/*w* of compound; ** Information not given.

## Data Availability

The data presented in this study are available on request from the corresponding author. The data are not publicly available because the authors want to keep priority for conference presentations.

## Supplementary Materials

The following are available online at https://www.mdpi.com/article/10.3390/mi12060715/s1, Figure S1: Comparison between (a) the common gas dilution method and (b) the pulse dilution method., Figure S2: working principle of the µBTEX-1 (In’Air Solutions, Strasbourg, France) with (a) the sampling step and (b) the separation and detection steps, Figure S3: (A) Main dimensions (in mm) of the gas micromixer (Reproduced with permission from Noël, F.; Serra, C.; Le Calvé, Micromachines; published by MDPI, 2019), channels’ depth was 1 mm, (B) schematics of the parts manufactured using a 1.5 milling tool (green) and a 0.5 mm milling tool (red), Table S1: List of targeted concentrations and those generated ranging between 3.7 and 100 ppb for 6 series of measurements performed from either a dilution bench validated in the laboratory or the new microfluidic generator of gas mixtures, Figure S4: Total BTEX peak area on a logarithmic scale vs. time obtained with the gas train generator coupled to a 4-stage micromixer for a generated gas mixture of 50 ppb at different flow rates, i.e., 12.5, 25 and 50 NmL min^−1^. A standard gas cylinder of BTEX at 1000 ppb of each compound was used. Other parameters were fixed at: *Q* = 25 NmL min^−1^; *P_pol_* = 200 mbar; *t_carrier_* = 1s, Figure S5: Total BTEX peak area vs. time obtained with the gas train generator coupled to a 4-stage micromixer for either a gas mixture of 50 ppb generated from a gas cylinder of BTEX at 1000 ppb of each compound, or pure nitrogen, both injected through the same fluidic circuit. Other parameters were fixed at: *Q* = 25 NmL min^−1^; *P_pol_* = 200 mbar; *t_carrier_* = 1 s, Figure S6: Relative pressure-time profiles in the cell after filling with the BTEX mixture (*P_cell_*) and after purge with pure nitrogen (*P_atm_*). Generation parameters were fixed at: *Q* = 25 NmL min^−1^; *P_po_*_l_ = 200 mbar; *t_carrier_* = 1 s and *C_pol_* = 50 ppb.
