# Supplementary material for: Experimental Validation of a Novel Generator of Gas Mixtures Based on Axial Gas Pulses Coupled to a Micromixer
Source: Micromachines (Basel). 2021 Jun 18;12(6):715. doi: 10.3390/mi12060715 (PMC8234469; doi:10.3390/mi12060715)
Supplement: Supplementary file 1 [file micromachines-12-00715-s001.zip › micromachines-1156025-supplementary.pdf]

Supplementary Materials

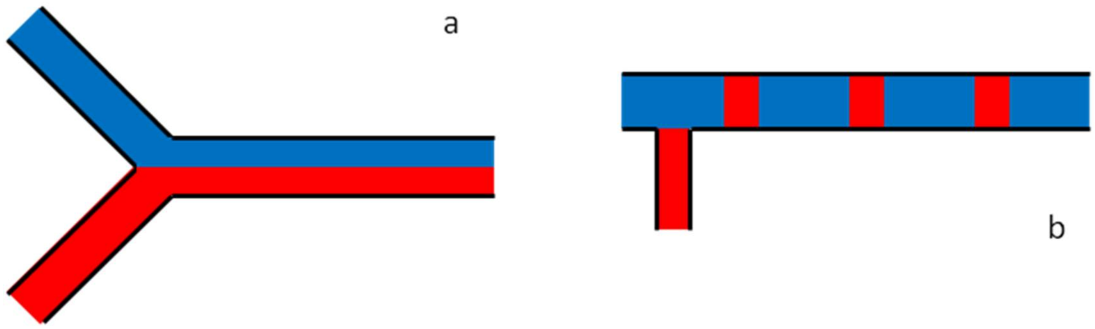

Figure S1. Comparison between a) the common gas dilution method and b) the pulse dilution.

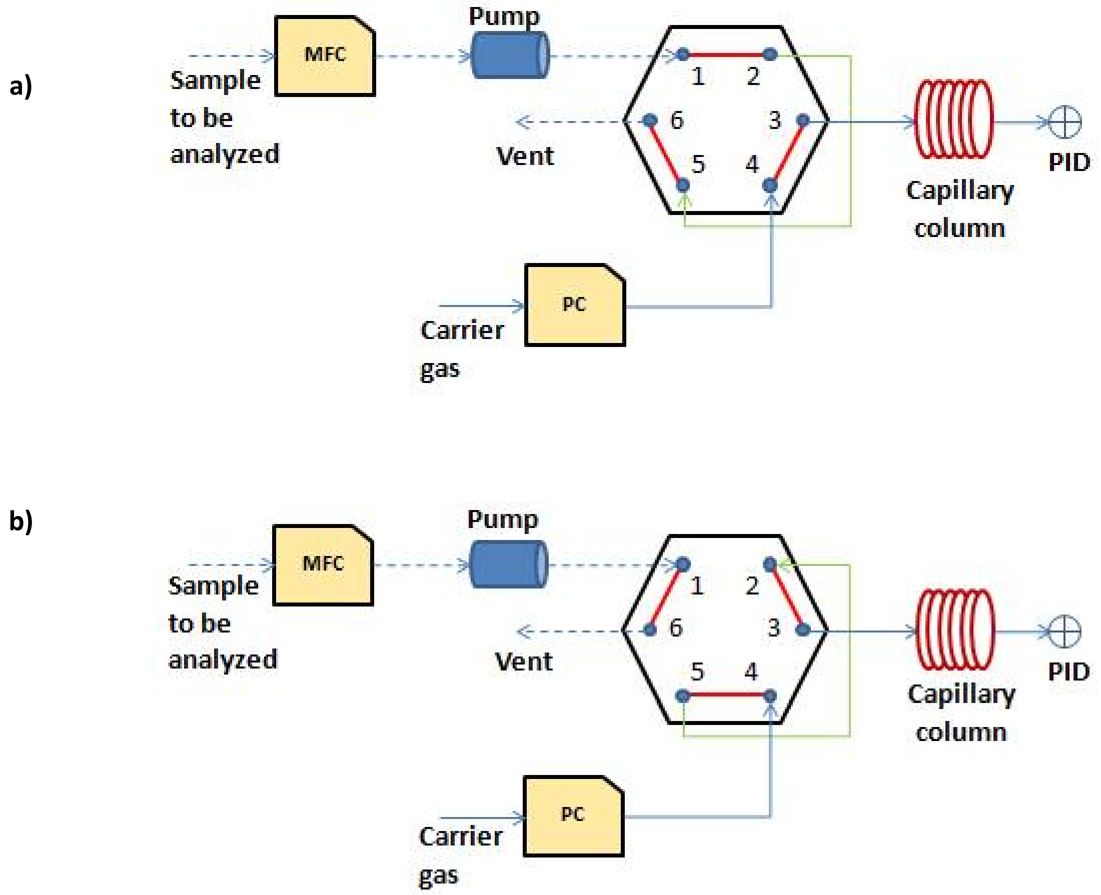

Figure S2. Working principle of the µBTEX-1 (In'Air Solutions, Strasbourg, France) with a) the sampling step and b) the separation and detection steps.

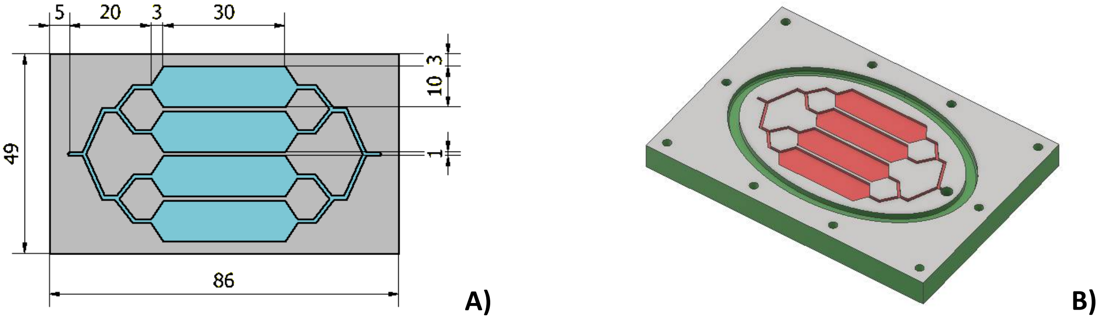

Figure S3. A) Main dimensions (in mm) of the gas micromixer (Reproduced with permission from Noël, F.; Serra, C.; Le Calvé, Micromachines; published by MDPI, 2019), channels' depth was 1 mm, B) schematics of the parts manufactured using a 1.5 milling tool (green) and a 0.5 mm milling tool (red).

Table S1. List of target concentrations and those really generated ranging between 3.7 and 100 ppb for 6 series of measurements performed from either a dilution bench validated in the laboratory or the new microfluidic generator of gas mixtures.

**Date**	**04/04/2019**	**17/04/2019**	**17/04/2019**	**04/04/2019**	**11/04/2019**	**12/04/2019**
**Target concentration (ppb)**	Dilution bench 100 ppb	New generator 100 ppb_1	New generator 100 ppb_2	Dilution bench 1000 ppb	New generator 1000 ppb_1	New generator 1000 ppb_2
**5**	5	3.7	3.7	-	-	-
**10**	10	8.1	8.4	10	15.3	12.3
**20**	20	17.7	17.7	20	16.6	16.0
**50**	-	-	-	50	41.7	40.6
**100**	-	-	-	100	87.8	86.4

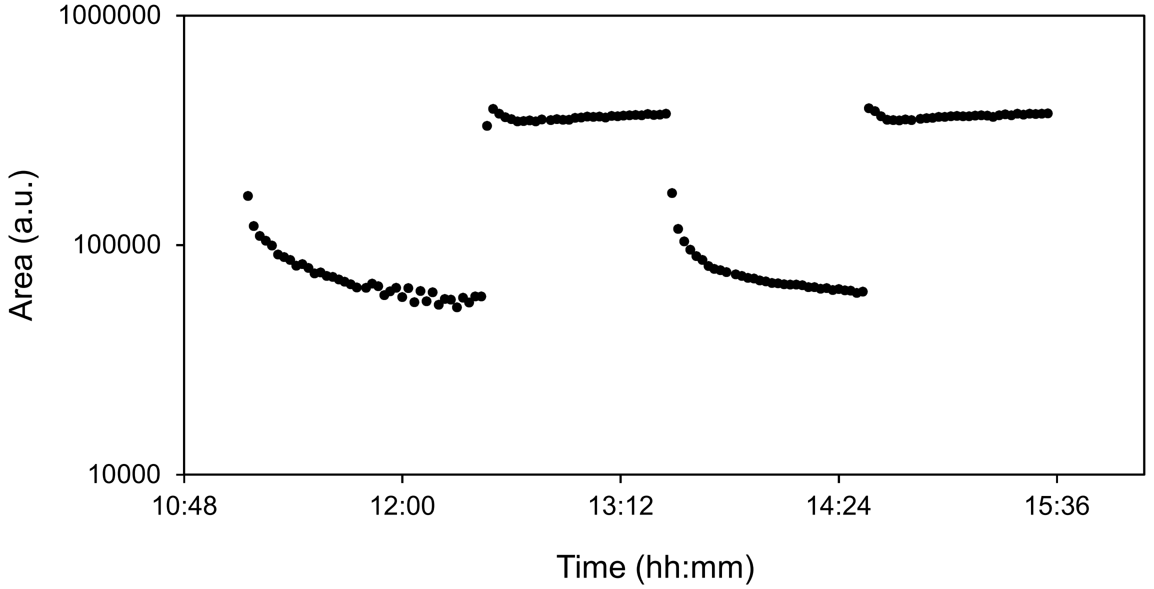

Figure S4. Total BTEX peak area on a logarithmic scale vs. time obtained with the gas train generator coupled to a 4-stage micromixer for a generated gas mixture of 50 ppb at different flow rates, i.e., 12.5, 25 and 50 NmL min^{-1}. A standard gas cylinder of BTEX at 1000 ppb of each compound was used. Other parameters were fixed at: Q = 25 NmL min^{-1}; P_{pol} = 200 mbar; $t_{carrier}$ = 1s.

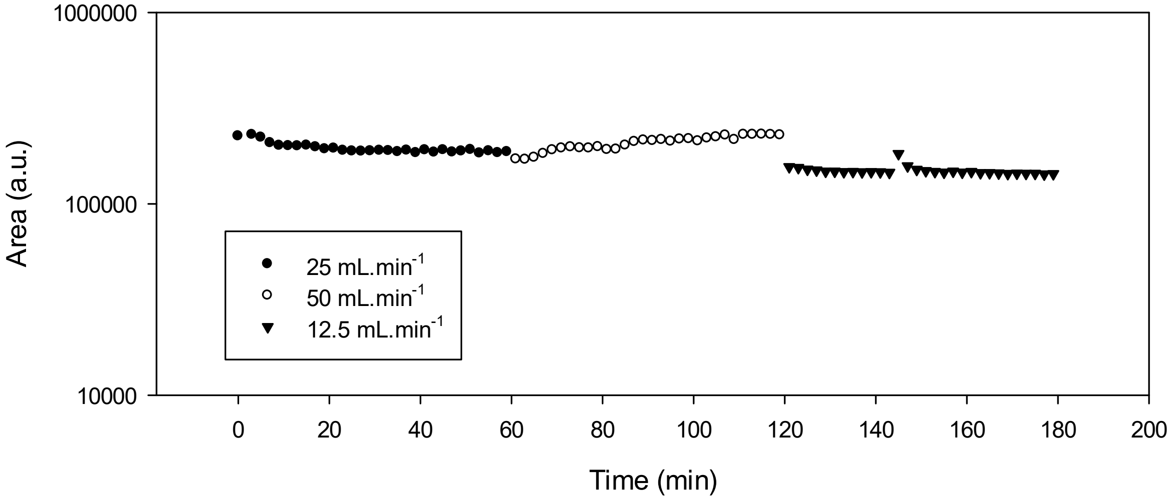

Figure S5. Total BTEX peak area vs. time obtained with the gas train generator coupled to a 4-stage micromixer for either a gas mixture of 50 ppb generated from a gas cylinder of BTEX at 1000 ppb of each compound, or pure nitrogen, both injected through the same fluidic circuit. Other parameters were fixed at: Q = 25 NmL min^{-1}; P_{pol} = 200 mbar; $t_{carrier}$ = 1s.

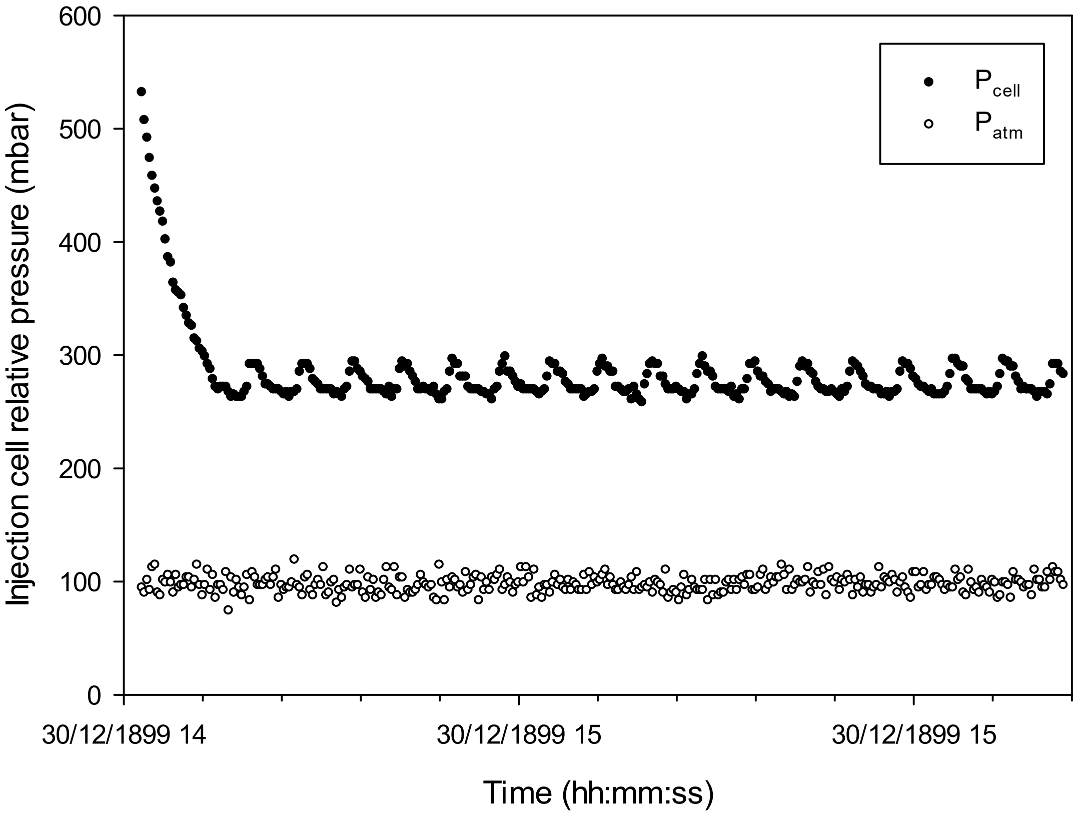

Figure S6. Relative pressure-time profiles in the cell after filling with the BTEX mixture (P_{cell}) and after purge with pure nitrogen (P_{atm}). Generation parameters were fixed at: Q = 25 NmL min^{-1}; P_{pol} = 200 mbar; $t_{carrier}$ = 1s; C_{pol} = 50 ppb.
